# Circulating Bubbles in Decompression Sickness: Pathophysiology and Experimental Models

**DOI:** 10.3390/biom16091339

**Published:** 2026-09-15

**Authors:** Zequn Jin, Qi Zhu, Juan Zheng, Baoliang Zhu, Kun Zhang, Guoyang Huang, Xuhua Yu, Weigang Xu

**Affiliations:** Department of Diving and Hyperbaric Medicine, Naval Medical Center, Naval Medical University, Shanghai 200433, China; jinzequn1011@163.com (Z.J.); chzhuqi@smmu.edu.cn (Q.Z.); zhengjuan81@smmu.edu.cn (J.Z.); zhubaoliang_001@163.com (B.Z.); 18301963086@163.com (K.Z.); huangswa@163.com (G.H.)

**Keywords:** decompression sickness, circulating bubbles, bubble nucleation, intravascular bubbles, endothelial dysfunction, microfluidic models

## Abstract

Decompression sickness (DCS) is a severe condition caused by rapid reductions in ambient pressure during diving, altitude exposure, or aerospace operations, with circulating gas bubbles serving as a central initiating event. Stable nanobubbles on active hydrophobic spots along the vascular lumen can grow under decompression-induced supersaturation, detach, and enter the circulation. Their subsequent behavior is shaped by blood flow, vessel geometry, and coagulation and may lead to thrombosis and microvascular stasis. DCS pathogenesis involves three linked interfaces: the blood–bubble interface activates coagulation and complement; the bubble–endothelium interface causes mechanical injury, endothelial dysfunction, oxidative stress, and cell death; and the endothelium–immune interface, which promotes neutrophil recruitment and NETosis, driving sterile inflammation and microthrombosis. Experimental approaches reproduce different stages of this pathogenic sequence and can be broadly distinguished according to the origin of the bubbles. Whole-animal decompression models and whole-blood decompression systems incorporate pressure reduction or gas supersaturation and can therefore generate bubbles within the experimental system. Many endothelial-cell, vessel-based, and microfluidic platforms use pre-existing or externally generated gas bubbles that are introduced directly into the experimental system. These directly introduced-bubble models do not reproduce decompression-induced bubble genesis; however, they provide precise control over bubble size, flow, vascular geometry, and cellular interactions. They are therefore valuable for dissecting post-formation processes shared by decompression-generated and directly introduced intravascular bubbles, including bubble transport, vascular retention, endothelial injury, thrombosis, and inflammation. Recognizing this distinction in bubble origin is essential for defining the translational relevance and limitations of individual experimental models.

## 1. Introduction

Decompression sickness (DCS) is a potentially life-threatening condition arising from a rapid reduction in ambient pressure, encountered in diving, high-altitude or aerospace operations [1]. The prevailing view is that DCS results from supersaturation of inert gases, mainly nitrogen, in body fluids and tissues, leading to the formation of intravascular and extravascular bubbles [1,2]. Although timely recompression and oxygen therapy are effective treatments for DCS, its clinical manifestations vary widely, ranging from mild symptoms such as joint pain and skin rash to severe neurological deficits, cardiopulmonary impairment, and shock [2]. Understanding how circulating bubbles progress from a physical phenomenon to triggers of complex systemic pathology remains a central challenge in diving and hyperbaric medicine [1].

Over the past decades, research has established that circulating bubbles trigger secondary responses including inflammation, coagulation, complement activation, and endothelial dysfunction [2]. At the blood–bubble interface, plasma proteins rapidly adsorb onto the bubble surface to form a “protein corona”, which activates platelets and leukocytes; these activated cells in turn release pro-inflammatory and pro-thrombotic mediators [3,4,5]. Meanwhile, at the bubble–vessel wall interface, direct contact between circulating bubbles and the endothelium imposes mechanical stress and deformation, leading to glycocalyx disruption, endothelial activation, and upregulation of adhesion molecules such as ICAM-1 and E-selectin [6,7,8]. This further promotes leukocyte adhesion, transendothelial migration, and loss of vascular integrity [7]. Together, these dual interfacial events amplify sterile inflammation, microvascular thrombosis, and endothelial dysfunction. Thus, explaining DCS requires moving beyond the physical dynamics of bubble formation to dissect the molecular and cellular interactions between bubbles, blood, and the vascular endothelium.

To systematically investigate these complex mechanisms and develop improved preventive or therapeutic strategies, a spectrum of experimental models has been established, ranging from whole-animal decompression models to isolated tissues, endothelial-cell cultures, whole-blood systems, and microfluidic vascular platforms. Importantly, these models reproduce different stages of DCS pathogenesis and should not be considered equivalent. In DCS, intravascular and extravascular bubbles arise primarily from dissolved inert gas that becomes supersaturated during or after a reduction in ambient pressure [2]. By contrast, intravascular gas embolism can also result from other mechanisms, including the direct introduction of gas during invasive medical procedures, and these conditions differ fundamentally from DCS in the origin of the bubbles despite sharing several downstream vascular consequences [4].

Accordingly, experimental models can be conceptually divided into two broad categories according to bubble origin. Models of decompression-generated bubbles incorporate a reduction in ambient pressure or dissolved-gas supersaturation, allowing bubbles to form within the experimental system and enabling investigation of nucleation, growth, and decompression susceptibility. In contrast, models using directly introduced bubbles employ pre-existing or externally generated gas bubbles that are introduced directly into blood, vascular tissues, endothelial cultures, or microfluidic channels [9,10,11,12]. These models primarily investigate bubble behavior and biological responses after the bubbles have entered the experimental vascular or cellular environment.

Models using directly introduced bubbles remain highly relevant to DCS because, once gas bubbles are present within the circulation, decompression-generated and directly introduced bubbles share many downstream biophysical and biological processes. These include bubble deformation and transport through branching microvessels, vascular retention or obstruction, disturbances in local blood rheology, bubble–blood and bubble–endothelium interactions, platelet and coagulation activation, endothelial injury, and thromboinflammation [4,9,10,11,12,13]. Therefore, rather than treating all experimental platforms as complete models of DCS, this review distinguishes models according to whether bubbles arise through decompression within the experimental system or are introduced directly from an external source. The latter models provide complementary tools for studying the post-formation behavior and biological consequences of intravascular bubbles without implying that they reproduce the decompression-dependent origin of DCS.

## 2. Generation of Circulating Microbubbles

### 2.1. Nucleation and Stabilization of Gas Nuclei

Homogeneous nucleation, in which bubbles form in a pure liquid without a pre-existing interface, requires extremely large tensile pressures and is therefore unlikely to account for physiological decompression bubbling [14,15]. Instead, DCS bubble formation is generally considered to depend on pre-existing gas nuclei and heterogeneous nucleation. Classical work proposed that hydrophobic crevices could trap and stabilize gas nuclei [16,17], and subsequent mathematical modeling showed how crevice geometry could support nucleus persistence during pressure changes [18]. Later experimental observations demonstrated that decompression bubbles can also develop at hydrophobic surfaces without a conventional macroscopic crevice, contributing to the concept of active hydrophobic spots on the vascular luminal surface [19,20,21,22].

AHS are composed of dipalmitoylphosphatidylcholine (DPPC), the main component of pulmonary surfactant, which leaks from the lung into the bloodstream and deposits on the vascular luminal surface [20,21]. Atomic force microscopy has directly visualized nanoscale gas domains on hydrophobic solid–liquid interfaces, providing physical support for the existence of persistent interfacial gas nuclei [15,21,23]. Although classical diffusion theory predicts rapid dissolution of very small free bubbles, interfacial nanobubbles can exhibit substantially greater stability because of surface attachment, contact-line pinning, local gas conditions, and interfacial effects [15,21,23]. These observations provide a physicochemical basis for considering stable surface-associated gas nuclei as potential precursors of decompression bubbles [19,20,21,22,24]. The mechanisms-likely involving contact line pinning, local gas supersaturation, and nanoscale roughness-are not fully understood [23,25]. Nevertheless, these stable nanobubbles are now considered the most plausible precursors of the pathological circulating microbubbles that grow during or after decompression [19,20,21,22,24].

In addition to hydrophobic interfaces, several other mechanisms have been proposed to promote or stabilize gas nuclei. Tissue elasticity creates a free-energy well that renders bubbles with radii below 0.8 μm or above 6 μm metastable, explaining the persistence of intratissue bubbles [26,27]. Yount’s variable permeability model proposes an elastic surfactant film that becomes gas-impermeable at high pressure, thereby preventing nucleus collapse [28]. Frictional nucleation from rapid separation of solid surfaces during muscle contraction or joint movement generates transient negative pressure and forms new nuclei, accounting for the increased DCS risk after physical activity [17,29]. Hyperbaric preconditioning can crush unstable nuclei and significantly reduce post-decompression bubbling, providing key evidence for the existence of gas nuclei [17,28,30]. In vivo, gas nuclei are maintained in a dynamic equilibrium balanced by generation and elimination, and interindividual differences in AHS number and activity account for the wide variation in decompression sensitivity and bubble loads [17,20,21,24].

### 2.2. Growth and Detachment of Gas Nuclei

Once ambient pressure drops and tissues become gas-supersaturated, stabilized gas nuclei enter a growth phase. The first step is initiation. For nanobubbles on AHS, activation to a visible size of approximately 0.1 mm is a slow and stochastic process, peaking around 45 min post-decompression and lasting for more than one hour [21,22,24,31]. This time course is broadly consistent with the typical early post-decompression onset of clinical DCS manifestations [2]. After crossing a critical size, the nucleus enters a rapid growth phase. However, because bubbles with radii between 0.8 μm and 6.0 μm are mechanically unstable according to the generalized Young-Laplace equation, the nucleus must grow beyond this range to survive [26,27,32]. The classical Epstein–Plesset equation predicts radius growth proportional to the square root of time, but in flowing blood the thinning boundary layer can lead to linear diameter growth [15,24,31]. Higher supersaturation favors bubble activation and growth [22,31]. Surface-active phospholipids such as DPPC may modify gas-nucleus behavior at hydrophobic vascular surfaces, although circulating DPPC concentrations do not reliably predict subsequent decompression bubbling [20,33]. However, subsequent studies found no significant correlation between circulating DPPC levels and the volume of bubble formation at AHS [33]. Multiple bubbles from the same or adjacent AHS compete for dissolved gas, slowing individual growth, whereas coalescence can cause sudden volume jumps [22,31].

Detachment occurs when driving forces exceed retaining forces. The main driving forces are buoyancy and blood flow shear stress; the primary retaining force is capillary adhesion at the anchoring site [21,34]. Mechanical disturbances, such as tapping or pulsatile flow, mainly affect the timing of detachment rather than the critical size at which a bubble detaches [21,34]. This explains why post-dive physical activity increases DCS risk: it causes early release of many small bubbles that are still growing, which can then act as nucleation sites for larger bubbles or directly obstruct microvessels [21,22].

### 2.3. Behavior of Circulating Bubbles in the Vascular System

Once detached, circulating bubbles are transported by blood flow, and their subsequent behavior is governed by the combined effects of vessel geometry, viscous stresses, interfacial forces, inertia, and buoyancy [15,35,36]. Importantly, these factors are relevant throughout the vascular tree rather than only in large vessels. Whole blood is a shear-thinning, non-Newtonian suspension whose apparent viscosity depends on shear rate, hematocrit, plasma viscosity, erythrocyte deformability, and erythrocyte aggregation [36,37]. These rheological properties influence the drag acting on a bubble, local velocity gradients, bubble residence time, and the shear stresses transmitted to the vascular wall [36]. The relative importance of viscous and interfacial forces is commonly represented by the capillary number, Ca = μU/σ, where μ is the characteristic liquid viscosity, U is the characteristic velocity, and σ is the gas–liquid interfacial tension [15,35,38,39]. Thus, changes in blood viscosity, flow velocity, or interfacial tension can shift bubble behavior between a surface-tension-dominated regime and a more deformable, viscous-force-dominated regime [15,35,38,39].

In larger vessels such as the vena cava and aorta, bubble motion is additionally influenced by inertia and buoyancy. In inclined or vertically oriented vessels, buoyancy can promote migration toward the upper wall and preferential entry into an upper branch, whereas its influence is substantially reduced when bifurcation geometry lies in a horizontal plane [35,40]. Pulsatile arterial flow can deform bubbles from spherical toward elongated shapes [36], while splitting or preferential branch entry at bifurcations depends on bubble size relative to vessel caliber, flow intensity, capillary number, Reynolds number, and bifurcation geometry [13,40]. Long bubbles may undergo different splitting behavior as Reynolds number and local flow conditions change [13]. In physiological vascular networks, curvature and bifurcation further generate secondary flows, recirculation zones, and spatially heterogeneous wall shear stress [41,42,43]. Consequently, viscosity and interfacial tension influence bubble transport even in relatively large vessels by modifying the balance between hydrodynamic deformation and capillary resistance.

Interfacial properties are particularly important because the gas–blood boundary is a dynamic biological interface rather than a clean gas–water surface. Interfacial tension contributes to the Laplace pressure within a bubble and therefore affects bubble shape, deformation, growth or dissolution, and the resistance of the interface to breakup [15,35]. Plasma proteins and lipids adsorb rapidly onto gas surfaces, altering the physicochemical properties of the interface and forming the biological coating or “protein corona” described for intravascular bubbles [35,44]. Adsorbed surface-active molecules can alter interfacial tension, while spatial variations in their concentration may generate Marangoni stresses that modify interfacial mobility and the surrounding flow field [35,38]. In computational models of nearly occluding vascular bubbles, soluble surfactants alter bubble motion and the distribution of shear stress along the vessel wall [38]. Experimentally, modification of the gas–blood interface with surfactants can also reduce embolic-bubble adhesion to the endothelium and attenuate endothelial injury [6]. Plasma composition and interfacial chemistry may therefore influence vascular bubble behavior through both physical and biological mechanisms.

These effects become particularly important in microvessels, where geometric confinement interacts strongly with blood rheology and interfacial tension. Bubbles that are small relative to the vessel lumen can move with the surrounding blood while undergoing deformation, whereas increasing confinement promotes axial elongation [36,38]. In the near-occluding regime, the non-Newtonian properties of blood and hematocrit-dependent changes in apparent viscosity affect bubble–wall separation, local shear stress, and bubble transit characteristics [36,45]. Computational studies further suggest that increasing hematocrit can amplify bubble-associated hydrodynamic stresses in small arteries, although this effect is substantially less pronounced in smaller arterioles, indicating that the consequences of altered rheology are vessel-size dependent [45]. In classical microchannel two-phase flow, an elongated gas bubble that occupies most of the channel cross-section and is separated from the wall by a thin liquid film is commonly described as a Taylor bubble or gas plug; in Taylor or slug flow, successive gas plugs are separated by liquid slugs [39,46]. The motion of these confined bubbles depends on bubble-to-vessel size ratio, apparent viscosity, interfacial tension, capillary number, and channel geometry [13,15,36,38,39].

These considerations are particularly relevant to the cerebral microcirculation, where cerebral arterioles generally range from approximately 10 to 100 μm in diameter [47]. In the cerebral microcirculation, the likelihood of vascular obstruction depends on the relationship between bubble size and vessel caliber, together with bubble deformability and local pressure and flow conditions [36,40,47]. However, neither geometry nor rheology alone determines whether a bubble becomes permanently arrested. Vessel bifurcations, bubble deformability, local flow, endothelial interactions, and thrombus formation all contribute to retention [36,40,47]. Recirculating flow around a retained bubble can promote local accumulation of fibrinogen and platelets, and progressive thrombus formation can disrupt the lubricating liquid film and increase resistance to bubble displacement [9]. Thus, microvascular obstruction reflects a coupled interaction among bubble size, vessel geometry, blood rheology, gas–blood interfacial properties, local hemodynamics, and biological responses.

**Figure 1:**
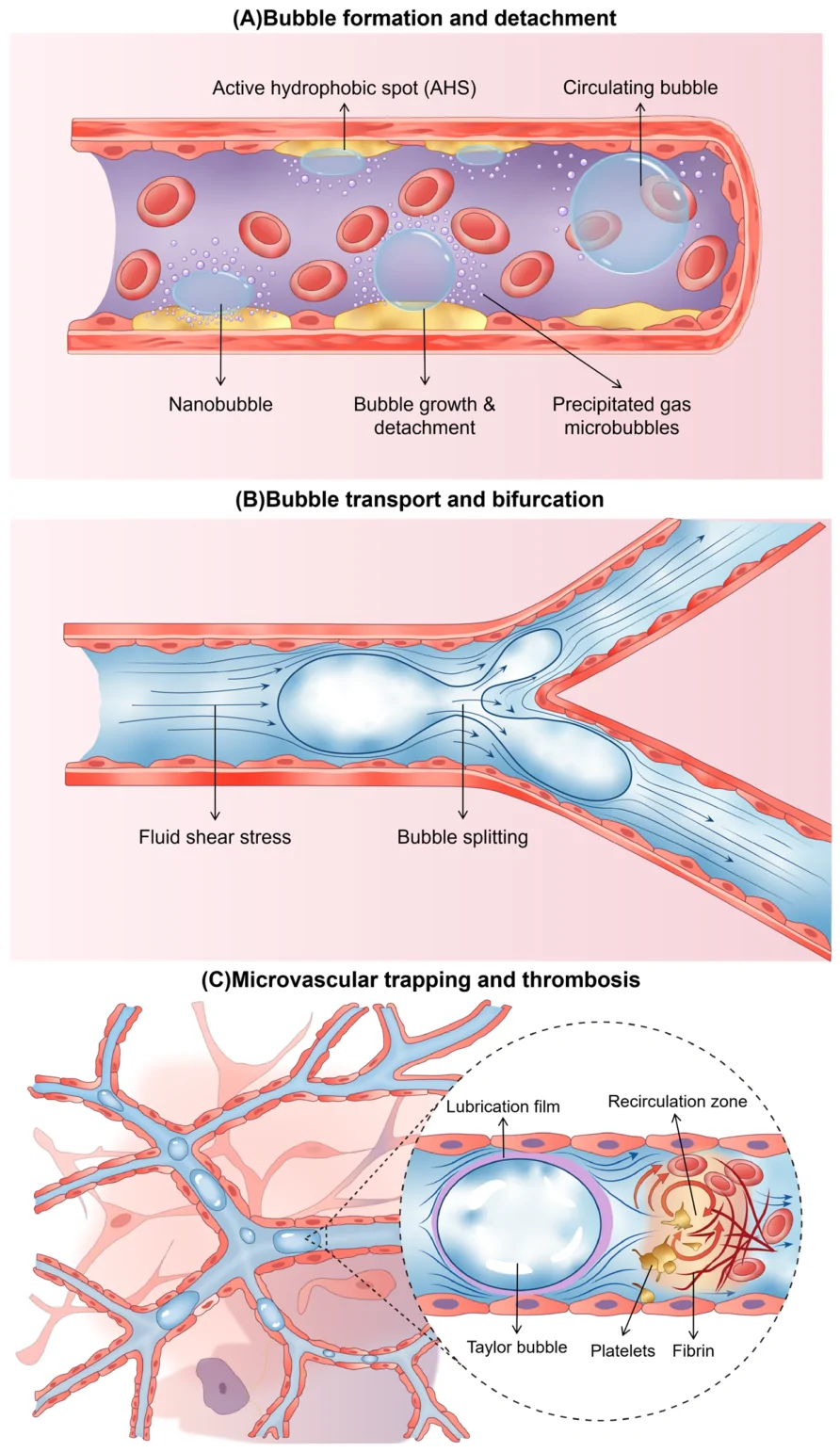
Formation, transport, and microvascular trapping of circulating bubbles in decompression sickness. (**A**) Stable nanoscale gas nuclei form on active hydrophobic spots (AHS) located on the vessel lumen surface. Supersaturated dissolved gas diffuses into surface-attached nanobubbles, driving progressive bubble growth. Once buoyancy and flow-induced shear forces exceed adhesive retention forces, bubbles detach from the vascular wall and enter the circulation. (**B**) Detached bubbles are transported by blood flow, where they deform into spherical or elongated shapes and may undergo splitting at vascular bifurcations under local hemodynamic forces. (**C**) In the microcirculation, increasing confinement can deform bubbles into elongated, plug-like morphologies separated from the endothelial surface by a thin liquid film. Local recirculating flow can promote platelet and fibrin accumulation, contributing to progressive bubble retention and microvascular obstruction.

In the venous system, venous bubbles are normally filtered by the lungs and exhaled, but bubbles smaller than 22 μm or overwhelming numbers can escape into the arterial circulation [4,35,48]. Arterialization of venous bubbles occurs through three main pathways. First, pulmonary filtration overload: the lung normally filters bubbles with a cutoff diameter of approximately 22 μm. When venous gas load exceeds capillary filtration capacity—for example, a single bolus > 20 mL or continuous infusion > 0.15 mL/kg/min for >30 min, bubbles can deform or rupture pulmonary capillaries and enter the systemic circulation. Massive venous gas can also cause right ventricular “air lock” and circulatory collapse [4,35,48]. Second, patent foramen ovale (PFO), which is present in approximately one quarter of adults, provides an important route for venous bubbles to enter the systemic arterial circulation and is associated with increased risk of selected forms of DCS [2,49]. Third, intrapulmonary arteriovenous anastomoses or fistulas (IPAVA/PAVF) may open during exercise, providing another right-to-left shunt pathway even in the absence of PFO [2,4]. The transition from bubble transport to pathological vascular obstruction is schematically illustrated in Figure 1.

## 3. Pathophysiology of Bubble-Induced Injury

### 3.1. Bubble–Blood Interface

Bubble motion in blood vessels or microchannels can substantially disturb local hemodynamics. Moving bubbles can generate marked spatial and temporal disturbances in local shear stress, pressure, flow separation, and recirculation [9,38,41]. Bubbles can also disrupt the normal distribution of erythrocytes, causing upstream accumulation, downstream rarefaction, and heterogeneous hematocrit distribution within bifurcating microvascular networks [11]. Bubble formation and dissolution may further alter gas concentrations and acid–base conditions in the local blood microenvironment [5]. Although changes in local osmolality have not been measured directly, associated alterations in ion concentrations could potentially modify the local osmotic environment. The air–liquid interface can also enrich reactive oxygen species and modulate oxidation reactions [50]. In pathological states involving intravascular bubbles, such as DCS, increased oxidative-stress markers have been detected in blood, including a 126% increase in ROS and a 23% increase in lipid peroxidation [51]. These findings indicate an association between intravascular bubble formation and blood oxidative stress.

Beyond these physical and chemical effects, intravascular bubbles can promote thrombosis through direct interactions with platelets, coagulation components, and the vascular endothelium. Direct bubble–blood contact promotes platelet adhesion to the gas–liquid interface and platelet–platelet binding [52,53], while bubble exposure can enhance thrombin generation [54]. Experimental decompression studies further support platelet involvement: bubble-induced platelet aggregation correlates with DCS severity in rats [55], and classic experiments demonstrated platelet consumption and microthrombus formation during decompression [56]. More recent ex vivo and in vivo studies show that intravascular gas exposure can activate hemostatic and inflammatory pathways concurrently [3,5]. In parallel, bubble adhesion and prolonged bubble–endothelium contact can damage the endothelial surface [6,52], and decompression-related bubble detachment, bubble-induced mechanotransduction, and abnormal shear stress can further impair endothelial integrity [57,58,59]. These changes favor local platelet adhesion and coagulation. Activated platelets, in turn, provide phosphatidylserine-rich procoagulant surfaces that support thrombin generation and fibrin formation, while thrombin further promotes platelet activation and stabilization of platelet–fibrin thrombi [60].

The composition of bubble-associated thrombi is likely to be strongly influenced by the local hemodynamic environment. Under higher-shear arterial conditions, thrombi are generally enriched in platelets and fibrin and have traditionally been described as “white” thrombi, whereas thrombi formed under lower-flow or stagnant conditions contain relatively more fibrin and incorporated erythrocytes and are commonly termed “red” thrombi [60,61]. These categories reflect relative differences in composition rather than strictly distinct thrombus types, because platelets, fibrin, erythrocytes, and leukocytes may coexist within the same thrombus [61]. This distinction is particularly relevant to a retained intravascular bubble, around which markedly heterogeneous flow conditions may develop. Recirculating and low-velocity flow near the bubble tail promotes local accumulation of fibrinogen, platelets, and other blood components [9]. As clot formation progresses, platelet and fibrin deposition can disrupt the thin lubricating film between the bubble and vessel wall, markedly increasing resistance to bubble displacement and stabilizing vascular occlusion [9]. Thus, rather than representing a single uniform thrombus phenotype, bubble-associated thrombi may contain variable proportions of platelet-rich and fibrin/erythrocyte-rich components depending on local shear, vessel geometry, endothelial injury, and the duration of bubble retention [4,9,60,61]. Direct histological characterization of these compositional phenotypes specifically in DCS remains limited.

A parallel pathological cascade is complement activation. Experimental studies using human whole blood and in vivo gas embolism models have demonstrated substantial C3 activation after blood–bubble exposure, together with generation of complement activation products and downstream inflammatory and hemostatic responses [3,5]. The alternative pathway amplification loop can further propagate C3 activation at blood-contacting interfaces [62]. C5a and other complement-derived inflammatory signals promote leukocyte recruitment and contribute to systemic inflammatory injury [3,5,62].

### 3.2. Bubble-Induced Endothelial Injury

Endothelial injury begins with direct physical forces. When a bubble detaches from an active hydrophobic spot on the vessel wall, the detachment force can tear the underlying endothelial cell membrane, causing focal endothelial denudation and exposure of the subendothelial elastic lamina as the initial physical event [57]. This process is accompanied by bubble–endothelium adhesion mediated by plasma proteins adsorbing onto the bubble surface and interacting with the endothelial glycocalyx [6,58]. As bubbles move through the microcirculation, they generate extreme spatiotemporal shear stress gradients, including solitary wave-like stress distributions and rapid sign reversal, subjecting endothelial cells to alternating compression and stretch. This mechanical impact is most severe in arterioles and can directly cause membrane fatigue and mechanical injury [9,41,57]. Bubble rupture also produces supersonic microjets and shock waves, imposing instantaneous high-amplitude forces on adjacent endothelial cells, leading to membrane perforation and cell damage [63].

These mechanical stimuli are converted into intracellular biochemical signals via mechanotransduction pathways. Bubble contact first activates mechanoreceptors on the endothelial membrane, for example through interaction between the gas–liquid interface and heparan sulfate in the glycocalyx, which pulls on the transmembrane protein syndecan and activates TRPV channels via the actin cytoskeleton, mediating extracellular calcium influx as an initial signaling event [6,58,64]. Calcium influx further activates multiple downstream pathways, including calcium-independent pathways involving PKCα that lead to loss of mitochondrial membrane potential and mitochondrial dysfunction, as well as activation of Rho kinase (ROCK) and inhibition of flippase, resulting in phosphatidylserine externalization and cytoskeletal rearrangement, thereby inducing endothelial microparticle (EMP) release [64,65].

The subsequent endothelial dysfunction has several interconnected manifestations. Bubbles directly disrupt tight-junction proteins such as ZO-1 and adherens junctions, leading to reduced transendothelial electrical resistance and increased vascular permeability; in the lung, this is reflected by an increased wet-to-dry ratio and tissue edema [7,66,67,68,69]. Bubble exposure also rapidly upregulates endothelial P-selectin. Activation of NF-κB further increases the expression of ICAM-1, VCAM-1, and E-selectin, thereby promoting leukocyte, particularly neutrophil, rolling, adhesion, and transendothelial migration [2,7,66]. Extravascular neutrophils release neutrophil extracellular traps (NETs) enriched in citrullinated histone H3 (Cit-H3) and myeloperoxidase (MPO), while intravascular fibrin networks entrap activated platelets and erythrocytes. Together, these processes amplify thromboinflammatory microvascular occlusion [2,7,66]. Oxidative stress further contributes to endothelial dysfunction. Bubble contact increases mitochondrial superoxide production, which reacts with nitric oxide to form peroxynitrite and thereby reduces NO bioavailability. At the same time, increased production of vasoconstrictors such as endothelin-1 contributes to vasomotor dysfunction [66,70,71]. Excessive mechanical and oxidative stress may ultimately induce endothelial apoptosis and pyroptosis [63,68,72,73]. The TXNIP/NLRP3/GSDMD pathway has been proposed as a potential mechanism linking oxidative stress to endothelial pyroptosis, although direct validation in bubble-exposed endothelial cells remains necessary [73]. Shock waves generated by bubble rupture may also activate the NLRP3 inflammasome and promote pyroptotic injury [63]. Autophagy appears to have a biphasic role: moderate activation is protective, whereas excessive activation promotes apoptosis [72].

**Figure 2:**
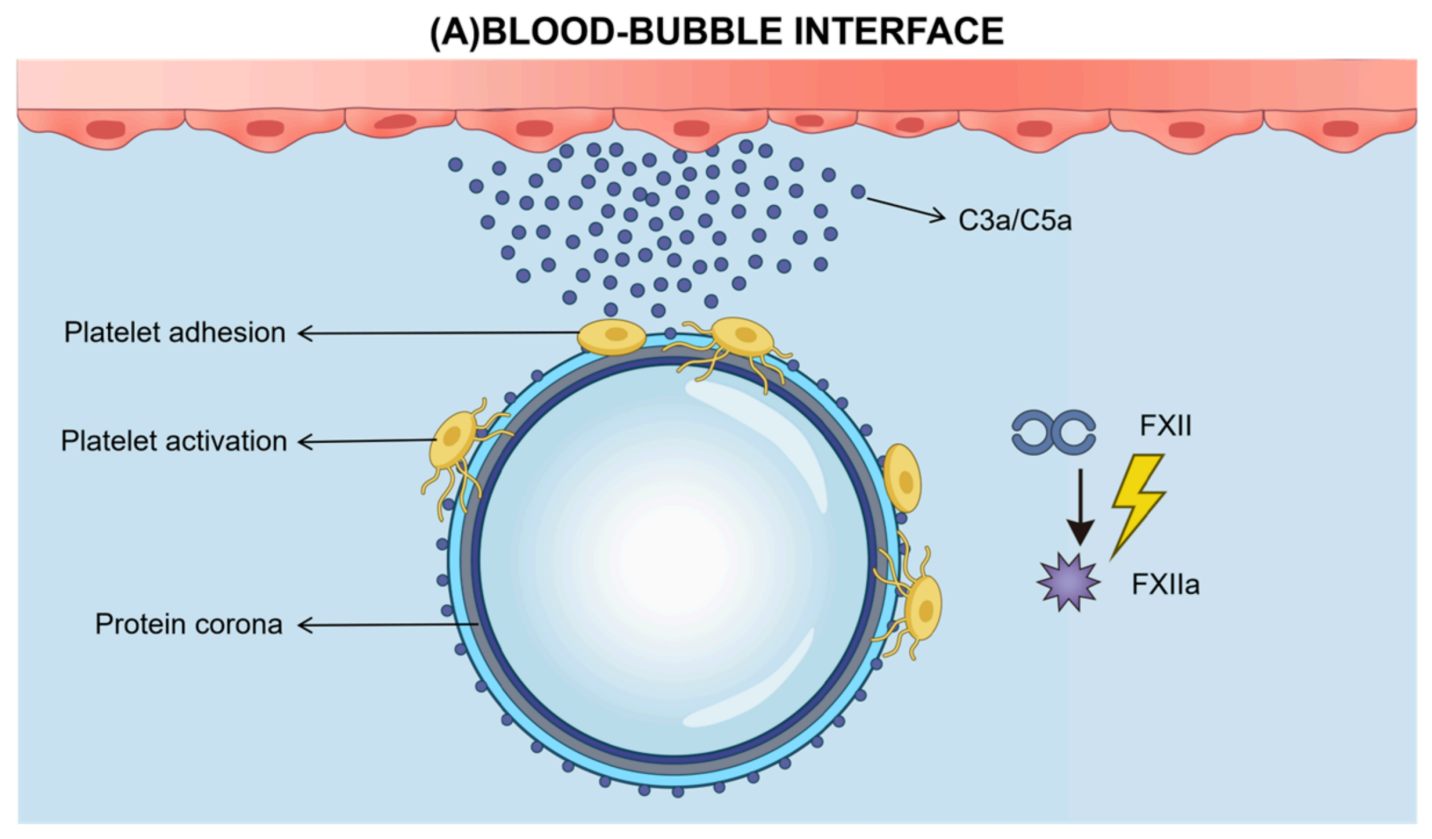

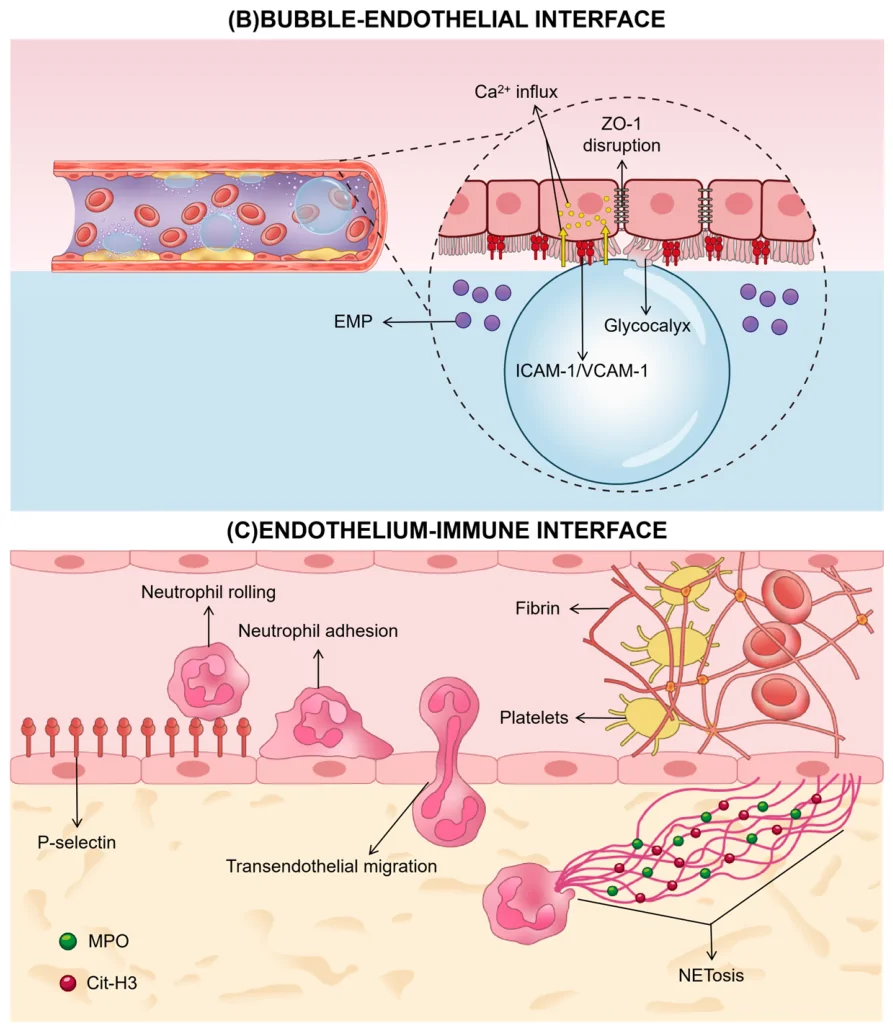
Bubble-driven thromboinflammatory cascades in decompression sickness. (**A**) At the blood–bubble interface, plasma proteins rapidly adsorb onto the gas surface to form a multilayered protein corona. Bubble exposure promotes complement activation with release of C3a and C5a, while factor XII (FXII) is converted to activated FXIIa, initiating contact coagulation. Platelets adhere to the bubble surface and undergo activation-associated shape change with pseudopod formation. (**B**) Direct contact between the bubble and the endothelial monolayer compresses the glycocalyx, elevates intracellular Ca^2+^, disrupts tight junctions such as ZO-1, and increases endothelial expression of ICAM-1 and VCAM-1. Endothelial microparticles (EMPs) are shed into the lumen as a consequence of barrier injury. (**C**) Activated endothelial cells upregulate P-selectin, promoting neutrophil rolling, adhesion, and transendothelial migration through PSGL-1-dependent interactions. Extravascular neutrophils release neutrophil extracellular traps (NETs) enriched in citrullinated histone H3 (Cit-H3) and myeloperoxidase (MPO), while intravascular fibrin networks entrap activated platelets and erythrocytes, amplifying thromboinflammatory microvascular occlusion.

These microparticles are not only markers of endothelial injury but also key pathogenic mediators. More recent studies extend this concept by showing persistent elevations of inflammatory extracellular vesicles and microparticles after diving or decompression, together with neutrophil activation and neurovascular inflammatory responses [74,75,76]. In murine DCS models, decompression-associated microparticles can contribute to persistent neuroinflammation and functional impairment, and recent evidence implicates endothelial CD36 as one mediator of microparticle-induced vascular and neuroinflammatory injury [75,76]. In particular, bubble-induced endothelial microparticles can be taken up by healthy endothelial cells, transmitting injury signals that reduce cell viability, increase apoptosis, exacerbate inflammation, and disrupt barrier function [68]. Endothelial activation and microparticles also trigger formation of platelet–neutrophil aggregates and neutrophil activation, which are critical for tissue injury. Activated neutrophils release reactive oxygen species, proteases, and neutrophil extracellular traps, directly damaging the endothelium through a process regulated by the P-selectin/PSGL-1/NOX2/PAD4 axis [2,7,77,78]. Together with bubble-induced platelet activation, complement cascade, and coagulation, these endothelial-derived factors amplify vascular leakage, microthrombosis, and ultimately the tissue injury characteristic of DCS [2,7]. The mechanism of the coagulation-inflammatory cascade and endothelial injury triggered by circulating bubbles is shown in Figure 2.

## 4. Experimental Models of Decompression-Generated and Directly Introduced Bubbles

Experimental models used to investigate circulating gas bubbles differ fundamentally in how the bubbles are generated. Whole-animal decompression models retain the physiological context in which reduction in ambient pressure produces tissue inert-gas supersaturation and subsequent bubble formation [2,66,79]. In contrast, many endothelial-cell, vessel-based, and microfluidic models use bubbles that are generated externally and introduced directly into the experimental system [9,10,11,12,80]. These two approaches address complementary rather than interchangeable questions. Accordingly, the following sections evaluate each model according to bubble origin and the subsequent physical or biological processes that it can reproduce.

### 4.1. In Vivo Animal Models

Animal models of DCS differ not only among species but also according to the pressure profile, decompression rate, and outcome measures used; therefore, phenotype labels should be interpreted as protocol-dependent experimental characteristics rather than fixed or mutually exclusive properties of a species [79,81,82,83]. Clinically, spinal DCS refers to neurological DCS in which manifestations are attributable predominantly to spinal cord involvement, including motor weakness or paralysis, sensory or proprioceptive deficits, and autonomic dysfunction [2]. Experimentally, a spinal DCS phenotype is identified by neurological signs such as hindlimb weakness or paralysis, reduced Tarlov scores, abnormalities in spinal evoked potentials, and/or histopathological evidence of spinal cord injury [81,82,83]. In contrast, the term “multisystem DCS phenotype” is used in this review as an operational experimental descriptor rather than a formal clinical subtype; it denotes concurrent measurable manifestations involving several organ systems, for example spinal/neurological, cutaneous, cardiopulmonary, hematological, or inflammatory injury [2,81]. These phenotypes can overlap. Accordingly, the phenotype designation in Table 1 indicates what has been reproducibly demonstrated or emphasized under representative experimental protocols, whereas “typical experimental applications” indicates the research questions for which each model is particularly useful.

Rodents, particularly Sprague–Dawley rats, are widely used in DCS research because of their low cost, well-established molecular toolkit, and suitability for relatively large experimental cohorts. Under commonly used decompression protocols, rats reliably reproduce circulating bubble formation together with endothelial, cardiopulmonary, inflammatory, and musculoskeletal injury, whereas sustained spinal paralytic DCS is less consistently reproduced [66,71,79,84]. Dedicated high-pressure and rapid-decompression protocols can induce thoracolumbar spinal cord injury and occasional paraplegia, although the effective severity window is relatively narrow because more aggressive exposures increase systemic morbidity and mortality [83]. Rats are therefore particularly useful for mechanistic studies of bubble burden, endothelial dysfunction, oxidative stress, inflammation, and tissue injury, while spinal DCS studies require more specifically optimized exposure protocols [66,71,79,83,84].

New Zealand white rabbits provide an intermediate-sized model that combines relatively convenient handling with sufficient body size for repeated blood sampling and physiological assessment [82]. They are particularly useful for spinal DCS research because representative decompression models reproducibly produce hindlimb motor impairment, reduced Tarlov scores, and histopathological spinal cord injury [82]. However, the phenotype is not exclusively neurological: pulmonary abnormalities, hematological and coagulation changes, and venous bubble formation can also be assessed, and recent work has established ultrasound-based bubble monitoring in this model [82,85]. Rabbits are therefore well suited to studies of spinal neurological injury, longitudinal biomarkers, bubble quantification, and neuroprotective interventions.

Bama miniature pigs are valuable large-animal models because their cardiovascular, respiratory, cutaneous, and spinal characteristics are more comparable with those of humans than are those of small laboratory animals, while their sparse hair facilitates direct assessment of cutaneous manifestations [81]. Importantly, porcine DCS models can reproduce spinal neurological injury together with substantial venous bubble loads, cutaneous lesions, pulmonary abnormalities, and hematological and coagulation changes [81]. Thus, the term “multisystem phenotype” reflects the ability of this model to reproduce several manifestations within the same animal and does not imply an inability to model spinal DCS. Bama miniature pigs are consequently useful for both focused spinal studies and integrated preclinical assessment of preventive or protective interventions, although their high cost and relatively small feasible cohort sizes limit large-scale experiments [81,86]. Overall, rats, rabbits, and Bama miniature pigs provide complementary experimental platforms spanning mechanistic small-animal studies, spinal and longitudinal assessments, and larger-animal translational research. Their relative strengths, limitations, representative phenotypes, and typical applications are summarized in Table 1.

### 4.2. In Vitro and Ex Vivo Models

In vitro and ex vivo models provide greater experimental control than whole-animal systems, but their relevance to DCS depends critically on whether they reproduce bubble formation itself. Only a limited number of platforms directly incorporate decompression or a reduction in ambient pressure. Whole-blood decompression systems, for example, expose blood to controlled pressure reduction and can therefore be used to investigate decompression-induced bubble nucleation and growth [63]. These systems are particularly relevant to the physical genesis of DCS bubbles, although they do not reproduce tissue inert-gas kinetics, intact vascular geometry, or systemic physiological responses.

In contrast, many endothelial-cell, isolated-vessel, and microfluidic studies introduce preformed or mechanically generated bubbles into the experimental system [9,10,11,12,80]. Bento et al. examined the effects of moving bubbles on erythrocyte distribution and local blood rheology [10,11]. Li et al. investigated the transition from bubble transport to vascular obstruction and demonstrated the contribution of flow recirculation and thrombus formation to bubble arrest [9]. Mardanpour et al. introduced gas through defined microfluidic inlets to investigate bubble persistence, transport, and arrest in networks mimicking the microvasculature [12]. These platforms should therefore be regarded as models using directly introduced bubbles rather than as models of decompression-induced bubble genesis. Nevertheless, they reproduce important physical and biological processes that also occur after decompression-generated bubbles enter the circulation and therefore provide valuable mechanistic information on bubble transport, deformation, vascular retention or obstruction, erythrocyte redistribution, thrombosis, and bubble–vascular interactions [4,9,10,11,12,35]. Cell-based models represent the most accessible and widely used in vitro platforms. Rat pulmonary microvascular endothelial cells (PMVECs), human umbilical vein endothelial cells (HUVECs), and human pulmonary microvascular endothelial cells (HPMECs) are commonly used because of their relevance to pulmonary and systemic vascular injury in DCS [7,64,68]. In static co-incubation systems, bubble diameter, gas volume, and exposure duration can be controlled, while outcomes such as intracellular Ca^2+^ signaling, EMP release, autophagy, and cell viability can be quantified. These studies have shown that bubble exposure promotes EMP release, activates Ca^2+^-dependent Rho/ROCK signaling and cytoskeletal rearrangement, and exerts biphasic effects on autophagy [64,66,68,71,77]. The TXNIP/NLRP3/GSDMD pathway has also been proposed as a potential mechanism of oxidative-stress-related pyroptosis, although direct confirmation in bubble-exposed endothelial cells is still required [73]. A major limitation of static systems is the absence of physiological shear stress, which substantially influences bubble–endothelium interactions in vivo [63,80,87].

Within static endothelial-cell models, HBO refers specifically to experimental pretreatment or preconditioning rather than to therapeutic recompression of established DCS. HBO pretreatment has been reported to enhance endothelial resistance to subsequent bubble injury through ROS/AKT/HSF-1 signaling and upregulation of HSP27 and HSP40 [88]. Such models are useful for dissecting cellular protective mechanisms but do not reproduce the principal physical effects of clinical recompression on bubbles that have already formed. In established DCS, therapeutic HBO combines increased ambient pressure with a high inspired oxygen partial pressure, thereby reducing bubble volume, accelerating inert-gas elimination, and improving oxygen delivery to ischemic tissues [2,89,90,91]. These clinical mechanisms are discussed further in Section 6.

**Table 1:** Comparative features of animal models used in decompression sickness research.

Model	Strengths	Limitations	Representative Protocol	Representative DCS Phenotype(s)	Core Readouts	Typical Experimental Applications	Refs.
Sprague–Dawley rat	Low cost; mature molecular toolkit; suitable for relatively large cohorts and repeated decompression paradigms	Limited blood volume for longitudinal sampling; species differences from humans; stable spinal paralytic DCS is less consistently reproduced under commonly used protocols	7 ATA for 90 min followed by rapid decompression over 3–5 min for endothelial/bubble studies; alternative protocols include 600 kPa for 60 min followed by rapid decompression	Systemic/cardiopulmonary, endothelial, inflammatory, and musculoskeletal injury; spinal cord injury can be induced with specialized severe protocols but is less reproducible	Bubble load; endothelial markers (ET-1, ICAM-1, MDA); lung injury; vascular reactivity; skeletal-muscle CK-MM and TLR9–MyD88 signaling; neurological and spinal histology in dedicated protocols	Mechanistic studies of bubble burden, endothelial dysfunction, oxidative stress, inflammation, skeletal-muscle injury and decompression adaptation; selected spinal DCS studies with optimized protocols	[66,71,79,83,84]
New Zealand white rabbit	Intermediate body size and cost; superficial marginal ear veins permit repeated blood sampling; relatively high and reproducible susceptibility to spinal DCS; established ultrasound bubble assessment	Dense fur limits assessment of cutaneous DCS; environmental sensitivity; inter-animal variability; mortality increases with excessively severe protocols	500 kPa gauge for 60 min followed by decompression at 200 kPa/min	Spinal-predominant neurological phenotype, with accompanying respiratory/pulmonary and systemic hematological changes	Tarlov score; respiratory/Atkins score; EB bubble grading; spinal cord and lung histology; lung wet/dry ratio; hematology and coagulation	Spinal DCS and neuroprotection studies; ultrasound-based bubble quantification; longitudinal hematological/coagulation biomarkers; intervention assessment	[82,85]
Bama miniature pig	Cardiovascular, respiratory, cutaneous and spinal characteristics relatively similar to humans; sparse hair facilitates skin assessment; allows repeated physiological monitoring and blood sampling; strong translational value	High cost; small feasible cohort sizes; limited statistical power for incidence/mortality outcomes; DCS susceptibility may exceed that of humans under equivalent severe exposure	600 kPa absolute for 30 min followed by staged decompression over ~10 min	Multisystem phenotype including spinal neurological, cutaneous, cardiopulmonary, hematological and coagulation abnormalities	Bubble load; Tarlov score; spinal somatosensory evoked potentials; skin lesion score; pulmonary hemodynamics; platelet/coagulation indices; lung and spinal cord histology; endothelial/inflammatory markers	Spinal DCS studies and integrated multisystem assessment; preclinical evaluation of preventive interventions; physiological monitoring; translational assessment of candidate protective strategies	[81,86]

Table note: Abbreviations: DCS, decompression sickness; EB, Eftedal–Brubakk bubble grading; ET-1, endothelin-1; ICAM-1, intercellular adhesion molecule-1; MDA, malondialdehyde; CK-MM, creatine kinase-MM. “Representative DCS phenotype(s)” denotes manifestations reproducibly observed or specifically assessed under the cited experimental protocols and does not imply that a species is restricted to that phenotype. “Spinal DCS” refers to neurological DCS with objective evidence of spinal cord involvement; experimentally, this may include hindlimb motor dysfunction or paralysis, reduced Tarlov scores, abnormalities in spinal evoked potentials, and/or spinal cord histopathology. “Multisystem DCS phenotype” is used here as an operational experimental descriptor rather than a formal clinical subtype and denotes concurrent measurable involvement of multiple organ systems. “Typical experimental applications” describes the research questions for which each model is particularly suited and should not be interpreted as an exclusive DCS phenotype or as a single experimental endpoint.

Ex vivo vessel-ring assays are a well-established approach for evaluating vasomotor function. Pulmonary arteries or aortic rings isolated from rats are mounted in tissue baths, where endothelium-dependent relaxation to acetylcholine and endothelium-independent relaxation to sodium nitroprusside can be assessed [92]. This approach minimizes neurohumoral confounding and enables direct assessment of intrinsic vascular function. A vessel-segment perfusion system developed in our laboratory further enables controlled delivery of intravascular bubbles and quantitative assessment of bubble-induced endothelial dysfunction. Because these models preserve the intact vascular wall, including the endothelium, smooth muscle, and adventitia, both contractile and relaxant responses can be evaluated. Their limitations include simplified shear conditions and the frequent use of protein-free physiological buffers rather than whole blood. In addition to eliminating blood-cell and plasma-protein interactions, such media differ from blood in viscosity, shear-dependent rheology, and gas–liquid interfacial properties. Because these parameters influence bubble deformation, mobility, adhesion, and bubble-induced wall stress, bubble-perfusion results obtained in buffer should not be assumed to reproduce physiological vascular bubble dynamics unless fluid rheology and interfacial properties are appropriately characterized [35,36,37,38].

The human whole-blood decompression model involves exposing fresh anticoagulated whole blood to controlled vacuum or decompression. This approach permits direct visualization of bubble nucleation and growth together with subsequent blood-phase changes [63]. An important advantage is that it retains native erythrocytes, platelets, plasma proteins, lipids, and coagulation components and therefore more closely preserves physiologically relevant hematocrit, blood viscosity, and gas–blood interfacial composition than protein-free systems [35,37,63]. This makes the model particularly useful for investigating how donor-dependent blood properties influence decompression-induced bubble formation and subsequent bubble–blood interactions. Nevertheless, anticoagulation, temperature, sample handling, and the absence of physiological flow can themselves alter rheological or interfacial behavior. Moreover, the lack of endothelium and vascular geometry prevents direct reproduction of bubble–endothelium–blood interactions under physiological shear [63].

Microfluidic circulation models are powerful in vitro platforms that have developed rapidly in recent years. These devices typically consist of microchannels ranging from approximately 20 μm to several hundred micrometers in diameter. Depending on the experimental design, channels may be lined with human endothelial cells and perfused with culture medium, blood analogues, or blood under controlled flow conditions [12,93,94]. High-speed microscopy allows individual microbubbles, including bubbles of approximately 20 μm in diameter, to be tracked in real time [80,95]. Microfluidic platforms have been used to study bubble deformation and splitting at bifurcations and to assess the likelihood of bubble arrest using dimensionless parameters such as the Euler, Weber, and capillary numbers [9,12]. Physiological fidelity in these models requires control not only of channel dimensions and flow rate but also of the properties of the perfused fluid. Hematocrit, apparent viscosity or the adopted rheological model, erythrocyte deformability and aggregation, plasma-protein content, and gas–liquid interfacial tension can all alter bubble motion and the local stresses generated around an embolus [10,11,12,35,36,37,38,45]. These variables also determine dimensionless parameters such as Reynolds and capillary numbers; therefore, reproducing a physiological flow rate without matching viscosity and interfacial tension does not necessarily reproduce the corresponding physiological bubble-flow regime [15,35]. Microfluidic studies should consequently report, and where possible experimentally control, hematocrit, temperature, fluid viscosity or rheological constitutive model, plasma or protein composition, and interfacial conditions in addition to channel geometry and flow rate. This standardization is particularly important when results obtained with water, culture medium, plasma, or whole blood are compared across studies. In addition, such platforms can quantify the adhesion and aggregation of blood cells, including red cells and platelets, at the bubble–endothelial interface and can assess vascular barrier function by measuring transendothelial electrical resistance (TEER) in real time with integrated electrodes [7,9,96]. Current microfluidic models remain highly reductive because many lack immune cells, complement, and complete three-dimensional tissue architecture [64,93]. Nevertheless, they provide access to fine-scale mechanical and cellular processes that are difficult to resolve in conventional animal experiments [80]. Among the platforms reviewed here, no single in vitro or ex vivo system currently reproduces the complete sequence of DCS from ambient-pressure reduction and tissue inert-gas supersaturation to bubble nucleation, bubble growth, vascular transport, endothelial injury, and downstream thromboinflammation. Whole-blood decompression systems reproduce pressure reduction and decompression-associated bubble formation but lack tissue inert-gas kinetics, vascular geometry, endothelium, and systemic physiological responses [63]. Conversely, most endothelial and microfluidic vascular models provide precise control of flow, vessel geometry, and bubble–vascular interactions but begin with externally generated or pre-existing bubbles [9,12,80]. Recent pressure-controlled organ-chip studies demonstrate that human vascularized tissues can be exposed to hyperbaric conditions followed by controlled decompression; however, the reported lung-on-chip model was designed primarily to examine dissolved-gas and immune responses rather than decompression-induced bubble formation [97]. A separate pressure-actuated vascular microfluidic platform demonstrated pressure-induced bubble generation and quantified the effects of channel geometry and fluid viscosity, but it used synthetic blood and did not reproduce tissue inert-gas supersaturation or the complete endothelial–immune response [98]. These complementary approaches demonstrate the feasibility of individual components while also defining the major technological gap that future integrated DCS models need to address [94]. Accordingly, the in vitro and ex vivo platforms summarized in Table 2 are classified primarily according to bubble origin. Decompression-generated bubble models produce bubbles within the experimental system through pressure reduction or gas supersaturation, whereas models using directly introduced or pre-existing bubbles begin with gas bubbles generated outside the relevant decompression process. The latter do not reproduce the decompression-dependent genesis of DCS bubbles but provide complementary platforms for investigating post-formation processes, including bubble transport, deformation, vascular retention or obstruction, blood–bubble interactions, endothelial injury, thrombosis, and inflammation.

## 5. Clinical Diagnosis of DCS and Translational Role of Experimental Models

### 5.1. Current Clinical Diagnosis of DCS

At present, DCS remains primarily a clinical diagnosis based on the relationship between a relevant decompression exposure and the subsequent development of compatible symptoms and signs [1,2,89]. The diagnostic assessment therefore begins with a detailed history of the dive or altitude exposure, including depth or pressure, duration, ascent profile, omitted decompression, repetitive exposure, symptom latency, and symptom evolution, followed by a focused physical and neurological examination [1,2,89]. This approach has the major advantage of being immediately available and of allowing treatment decisions to be made without waiting for ancillary testing, which is particularly important in serious neurological or cardiopulmonary DCS [1,2]. Its principal limitation is that many manifestations of DCS, including musculoskeletal pain, dizziness, paraesthesia, fatigue, and neurological symptoms, are not disease-specific and may overlap with barotrauma, immersion pulmonary edema, musculoskeletal injury, vestibular disorders, or unrelated neurological disease [1,2]. Consequently, no single symptom, laboratory test, or imaging finding currently serves as a definitive diagnostic reference standard for DCS [2,89].

Ancillary investigations can provide supportive information or help exclude alternative diagnoses, but their results must be interpreted in the clinical context. Doppler ultrasound and two-dimensional echocardiography can detect and semi-quantitatively grade post-decompression venous gas emboli (VGE), providing a non-invasive, repeatable measure of decompression stress [2,102]. However, circulating bubbles are not synonymous with clinical DCS: substantial VGE loads can occur in asymptomatic individuals, whereas clinically important DCS may occur with a low or undetected VGE burden. VGE grading is therefore valuable for decompression research, risk assessment, and comparison of decompression procedures, but it cannot independently confirm or exclude DCS in an individual diver [2,102]. Emerging non-ultrasound approaches are also being explored. For example, electrical-impedance spectroscopy has recently been evaluated in divers as a potential operator-independent method for monitoring post-dive decompression stress, although calibration and formal validation of diagnostic sensitivity and specificity remain necessary [103]. In neurological DCS, magnetic resonance imaging can demonstrate cerebral or spinal lesions and is useful when alternative structural diagnoses are being considered, but acute imaging sensitivity is limited, particularly for spinal cord DCS; consequently, a normal MRI does not exclude clinically significant disease [1,2]. Routine hematological, biochemical, coagulation, and arterial blood-gas measurements are principally used to assess disease severity, complications, or alternative diagnoses rather than to establish DCS itself [2].

A complementary research direction is the development of circulating biomarkers reflecting the vascular and inflammatory responses to decompression. Candidate signals include circulating microparticles or extracellular vesicles, endothelial microparticles, neutrophil activation, and markers of endothelial, oxidative, and inflammatory injury [7,51,66,68,74,75,76,77,78]. However, a major challenge is distinguishing pathological DCS from the physiological responses that also occur after uneventful decompression. For example, microparticle/extracellular-vesicle production and inflammatory activation can occur after diving even without clinically diagnosed DCS [74], indicating that their presence alone is insufficiently specific for diagnosis. Importantly, a human case–control study comparing divers with clinically diagnosed DCS with asymptomatic divers found substantially greater elevations of several phenotypically defined circulating microparticle populations and neutrophil myeloperoxidase expression in DCS [104]. Nevertheless, overlap between groups and dependence on the timing of blood sampling prevented these markers from being considered stand-alone diagnostic tests [104]. Thus, current biomarker evidence supports further development of multimarker and temporally resolved diagnostic approaches, but no circulating biomarker or biomarker panel has yet been sufficiently validated to replace clinical diagnosis [2,89,104].

### 5.2. How Experimental Models Can Support Diagnostic Development

Experimental models can contribute to diagnostic development without themselves constituting diagnostic tests. Their principal value is to provide controlled platforms in which candidate biomarkers or detection technologies can be related to a known decompression exposure, bubble burden, tissue injury, and disease phenotype. Whole-animal decompression models are particularly useful at the discovery and preclinical-validation stages because the initiating pressure reduction, subsequent bubble formation, clinical-like manifestations, and systemic biological responses occur within the same organism [66,71,81,82,85]. Serial sampling before and after decompression can therefore define the temporal trajectories of candidate markers and determine whether they correlate with bubble burden, neurological or cardiopulmonary severity, endothelial dysfunction, or tissue pathology [66,71,81,82,85]. Rabbit and porcine models additionally permit repeated blood sampling, ultrasound bubble grading, neurological assessment, and histopathological correlation, providing an opportunity to test whether a candidate marker tracks disease phenotype rather than decompression exposure alone [81,82,85]. Such experiments can identify promising marker combinations and optimal sampling windows for subsequent evaluation in human divers.

In vitro and ex vivo models address a different stage of this pipeline. Whole-blood decompression systems reproduce pressure reduction and decompression-induced bubble formation while retaining native blood cells and plasma proteins, making them useful for identifying blood-phase signatures associated directly with decompression and bubble genesis under controlled conditions [63]. Endothelial-cell and vessel-based models can then determine whether candidate endothelial markers, such as EMP release or changes in vascular function, arise causally from bubble–endothelium interactions rather than representing nonspecific systemic responses [64,68,92]. Microfluidic vascular models provide an additional level of mechanistic control by independently varying bubble size, frequency, flow, shear stress, vascular geometry, and blood composition while simultaneously measuring bubble arrest, platelet or fibrin deposition, endothelial permeability, and cellular responses [9,12,80,93,94,96,99,100,101]. These platforms are therefore well suited to determining the conditions under which a proposed biomarker or physiological readout is generated and to assessing its relationship to specific components of bubble-induced vascular injury. However, because most of these systems model only selected components of DCS, findings from them require confirmation in decompression-based animal models and ultimately in clinically characterized human cohorts.

A clinically useful diagnostic marker must ultimately distinguish DCS from both asymptomatic decompression responses and relevant differential diagnoses. Accordingly, translation should proceed from mechanistic discovery in controlled models to prospective human validation that includes symptomatic DCS cases and appropriately matched asymptomatic exposed divers [2,77,104]. Studies should incorporate standardized sampling intervals, detailed exposure profiles, serial clinical examination, and, where feasible, quantitative bubble assessment, while diagnostic performance should be evaluated using prespecified thresholds, sensitivity, specificity, and independent validation cohorts [104]. In this framework, experimental models contribute to diagnosis indirectly by identifying candidate markers, defining their biological origin and temporal behavior, and prioritizing combinations for clinical testing; they should not be regarded as evidence that any biomarker or technology is already validated for routine diagnosis of DCS.

## 6. Therapeutic Management of Decompression Sickness

Management of suspected DCS begins with early recognition, administration of the highest feasible inspired oxygen concentration, supportive care, and prompt consultation with a diving-medicine or hyperbaric specialist [1,2,89,90,91]. Breathing 100% oxygen at surface pressure lowers the alveolar inert-gas fraction toward zero and consequently reduces inert-gas tension in arterial blood, creating a diffusion gradient that promotes movement of nitrogen or other inert gases from supersaturated tissues and bubbles into the blood for pulmonary elimination [2,90]. Patients with acute DCS should generally be kept supine and unnecessary exertion should be avoided, while hydration should be maintained; isotonic, glucose-free intravenous crystalloid is appropriate when hypovolemia, dehydration, or hemodynamic compromise is present [2,89,90,91]. Serial neurological assessment is important because manifestations may evolve after presentation. Serious neurological, vestibular, or cardiopulmonary manifestations, progressive symptoms, or hemodynamic instability warrant urgent transfer for recompression, whereas management of selected mild and improving cases may be individualized in consultation with an experienced diving-medicine physician [1,2,91].

Recompression combined with hyperbaric oxygen is the definitive treatment for clinically significant DCS and acts through complementary physical and physiological mechanisms [1,2,89,90,91]. First, increasing ambient pressure directly decreases the volume of intravascular and extravascular gas according to Boyle’s law. For an idealized free gas bubble at constant temperature, increasing pressure from 1 ATA to 2.8 ATA would reduce its volume to approximately 36% of its surface-pressure volume, before accounting for gas diffusion, surface tension, and tissue constraints [89,90]. Bubble shrinkage can reduce mechanical distortion of tissues and decrease the length or degree of microvascular obstruction produced by intravascular bubbles [2]. Second, breathing 100% oxygen greatly lowers the inspired and alveolar partial pressure of inert gas. The resulting reduction in arterial inert-gas tension increases the diffusion gradient from tissues and bubbles to blood and ultimately to the lungs, thereby accelerating inert-gas washout and bubble resolution [2,90]. These two effects are synergistic: recompression immediately reduces bubble dimensions, while oxygen breathing favors continued elimination rather than renewed uptake of inert gas during treatment [2,90].

The elevated oxygen partial pressure during HBO also markedly increases dissolved oxygen in plasma and extends the effective diffusion distance of oxygen through tissues, which can support cellular oxygenation in vascular territories in which bubble obstruction, endothelial swelling, edema, or microthrombosis has impaired normal perfusion [2,89,90]. This mechanism is particularly relevant to neurological DCS, in which ischemia and microcirculatory compromise may persist even after the dimensions of the original bubbles have decreased [1,2]. Hyperbaric oxygen may additionally influence post-ischemic and inflammatory responses, although these biological effects are complementary to the primary physical effects of recompression, inert-gas elimination, and restoration of tissue oxygenation [2,89,90].

The U.S. Navy Treatment Table 6 (USN TT6), a standardized oxygen recompression protocol, is widely used for the initial treatment of serious DCS. The protocol begins at approximately 2.8 ATA and provides intermittent 100% oxygen breathing, with air breaks incorporated to reduce the risk of central nervous system oxygen toxicity [2,90,91]. Treatment should be initiated as early as practicable in serious DCS, because earlier recompression is generally associated with better neurological outcome; however, a prolonged delay does not establish a point beyond which recompression is necessarily ineffective, and patients with persistent compatible manifestations should still be evaluated for HBO [2,90]. Residual clinically significant manifestations after the first recompression may justify additional HBO sessions until recovery is complete or further sustained improvement is no longer observed [2,90]. Supportive management includes correction of hypovolemia and hypotension, maintenance of normothermia and appropriate neurological supportive care, and venous-thromboembolism prophylaxis in patients with substantial immobility or paralysis [2,89,91]. Maintaining euvolemia may also limit marked hemoconcentration, which increases apparent blood viscosity and could further compromise microvascular perfusion in the presence of bubble obstruction [2,37,45,91]. However, manipulation of hematocrit or viscosity is not an established disease-specific treatment for DCS, and fluid administration should remain directed toward correction of clinically relevant dehydration, hypovolemia, or hemodynamic compromise [2,91]. Modification of bubble interfacial properties is likewise mechanistically attractive but remains experimental. Surfactants have been shown in gas-embolism models to alter bubble hydrodynamics, reduce bubble–endothelium adhesion and endothelial injury, and reduce platelet–bubble interactions or thrombin generation [6,38,53,54]. These findings support the gas–blood interface as a potential therapeutic target, but no surfactant-based strategy is currently established for clinical treatment of DCS. Pharmacological adjuncts have a much weaker evidence base than recompression and oxygen. Non-steroidal anti-inflammatory drugs may reduce the number of additional recompression sessions in some patients but have not been shown to improve final neurological outcome, and no antiplatelet, anticoagulant, corticosteroid, or other mechanism-specific drug is established as routine therapy for DCS in the absence of another clinical indication [2,91]. Practical limitations of HBO include access to an appropriate chamber, evacuation delays, barotrauma, and oxygen toxicity; consequently, treatment decisions should integrate symptom severity, evolution, transport logistics, and specialist assessment rather than relying solely on bubble detection or symptom duration [1,2,90,91].

## 7. Future Perspectives and Discussion

Future DCS research should move beyond the use of individual models that reproduce isolated stages of disease and toward a coordinated multi-scale framework linking decompression physics to vascular and systemic injury. Whole-animal decompression models retain tissue inert-gas kinetics, physiological circulation, bubble formation, and clinically relevant neurological, pulmonary, and systemic phenotypes, but they provide limited control and visualization of individual bubble–vascular interactions and remain subject to species-specific differences [66,81,82,85]. Conversely, endothelial, vessel-based, and microfluidic models offer substantially greater spatial, temporal, and mechanistic control and can increasingly incorporate human cells and physiologically relevant vascular structures [80,94,96], but most currently reproduce only events occurring after bubbles have already formed. These model classes should therefore be regarded as complementary components of a translational pipeline rather than as competing or interchangeable representations of DCS.

A major future priority is the development of pressure-programmable, human-relevant experimental platforms capable of reproducing the DCS sequence within the same system. Ideally, such a platform would first permit controlled compression and inert-gas equilibration of blood and tissue-like compartments, followed by a defined reduction in ambient pressure that generates physiologically relevant supersaturation and in situ bubble nucleation. The same system should permit real-time observation of bubble growth, detachment, vascular transport, deformation, bifurcation, and arrest within perfusable endothelialized microvascular networks. Where feasible, it should also preserve interactions among blood cells, plasma proteins, coagulation factors, complement, and immune cells [5,7,9,63,94,96]. Recent studies already demonstrate separately that organ-on-chip devices can be exposed to controlled hyperbaric and decompression profiles and that pressure-based vascular microfluidic systems can generate and track gas bubbles [97,98]. However, these capabilities have not yet been integrated into a single model that reproduces the complete DCS cascade. Combining pressure control, humanized microvasculature, physiological perfusion, whole-blood components, and downstream inflammatory readouts would bridge current decompression-generated bubble models and directly introduced-bubble models within a single integrated experimental platform.

For diagnostic development, the principal opportunity offered by such integrated models is not direct diagnosis of DCS but the generation of temporally resolved relationships among decompression exposure, bubble formation, biological injury, and candidate biomarkers. Current clinical diagnosis remains dependent primarily on exposure history and compatible clinical manifestations, and neither VGE detection nor any circulating biomarker has sufficient sensitivity and specificity to independently confirm or exclude DCS [89,102,104]. Experimental platforms could help identify when candidate signals first emerge relative to bubble formation and determine whether they reflect physiological decompression stress or clinically relevant vascular injury. This distinction is essential because microparticles, extracellular vesicles, and inflammatory responses can also increase after diving in the absence of clinically diagnosed DCS [74], whereas more pronounced alterations have been reported in clinically diagnosed DCS [104]. Future animal studies should therefore combine serial blood sampling, quantitative bubble monitoring, neurological or cardiopulmonary phenotyping, and tissue pathology [81,82,85], while advanced microphysiological systems could incorporate continuous optical, electrical, and biochemical sensing to measure bubble dynamics, tissue oxygenation, endothelial-barrier function, and inflammatory signals in real time [94,105]. Candidate biomarker panels emerging from these platforms would then require prospective validation in well-characterized human cohorts before clinical implementation [89,102,104]. Emerging wearable or minimally operator-dependent physiological sensing approaches may provide complementary longitudinal measures of decompression stress, but they also require prospective clinical validation [103].

Therapeutic modeling should similarly distinguish prevention or preconditioning from treatment of established DCS. Current cellular studies can examine protective interventions administered before bubble exposure, including HBO pretreatment [88], but such models do not reproduce the principal physical effects of clinical recompression on bubbles that have already formed. A pressure-programmable full-sequence platform would permit bubbles to develop first and could subsequently apply defined recompression and hyperoxic treatment profiles, thereby reproducing the temporal sequence of clinical therapy [90,91,97]. This would allow simultaneous measurement of bubble shrinkage and clearance, restoration of tissue oxygenation, endothelial-barrier recovery, platelet and fibrin deposition, and persistence or resolution of inflammatory injury. The same platforms could then evaluate mechanism-based adjunctive interventions targeting the gas–blood interface, endothelial dysfunction, complement activation, platelet–bubble interactions, or neutrophil-mediated injury [5,6,7,53,54]. Importantly, such experimental treatments should be evaluated as potential adjuncts to, rather than replacements for, timely oxygen administration and recompression, which remain the established treatment for clinically significant DCS [90,91].

Prevention represents a further application of integrated modeling. Rather than evaluating decompression profiles solely by the presence or absence of post-decompression bubbles, future studies should determine how pressure history influences the complete sequence from supersaturation and bubble formation to vascular injury and clinical phenotype. Quantitative VGE assessment remains useful for decompression research but is an imperfect surrogate for disease [89,102]. Combining bubble measurements with endothelial, inflammatory, and functional endpoints in animal and humanized in vitro models may therefore provide more informative criteria for comparing decompression procedures [24,85,102]. Pressure-controlled microfluidic systems also provide an opportunity to systematically vary vessel geometry, fluid properties, pressure gradients, and decompression profiles while directly observing bubble formation and transport [98]. Ultimately, these experimental data could contribute to more individualized prevention strategies, but candidate algorithms or decompression procedures would still require validation under controlled animal conditions and, where appropriate, prospective human diving studies before routine implementation [2,89].

Rather than expecting a single experimental model to answer every question, future translation should use a staged validation strategy. Mechanistic hypotheses and candidate biomarkers or therapies can first be examined under precisely controlled conditions in whole-blood, endothelial, or vascular-chip systems. Effects on intact vascular function can then be evaluated in ex vivo vessels. Clinically relevant efficacy and safety should subsequently be tested in decompression-based animal models before human validation [63,80,81,82,92,94]. Standardization across these levels will be essential. Studies should report decompression profiles, gas composition, bubble burden, vascular geometry, blood or perfusate properties, sampling time, and predefined biological and functional endpoints so that results can be compared across laboratories. For diagnostic candidates, validation should emphasize sensitivity, specificity, temporal behavior, and the ability to distinguish symptomatic DCS from asymptomatic decompression responses [89,102,104]. For therapeutic candidates, both physical endpoints such as bubble clearance and biological endpoints such as endothelial, thrombotic, inflammatory, and neurological recovery should be assessed [90,91]. This multi-scale approach would allow experimental models to contribute not only to mechanistic understanding but also to the systematic development of diagnostic, therapeutic, and preventive strategies for DCS.

The long-term goal should therefore be the development of experimental systems that connect the physical origin of decompression bubbles with their downstream biological consequences rather than studying these stages in isolation. Pressure-programmable humanized vascular platforms, particularly when combined with whole-blood components, organ-specific tissues, and continuous multimodal sensing, offer a promising route toward this goal [94,96,97,98,105]. Such models will not eliminate the need for decompression-based animal studies or clinical validation, but they may provide the missing mechanistic bridge between pressure exposure, bubble formation, vascular injury, and clinically measurable outcomes. Establishing this bridge will be essential for converting knowledge of bubble physics and thromboinflammation into improved prevention, earlier recognition, and more precisely targeted treatment of DCS.

## 8. Conclusions

Decompression sickness is best understood as a continuum linking decompression-induced gas supersaturation and bubble formation to subsequent vascular obstruction and secondary biological injury. Circulating bubbles are not merely passive mechanical emboli. Their interactions with blood components and the vascular endothelium can initiate platelet and coagulation activation, complement signaling, endothelial dysfunction, oxidative stress, leukocyte recruitment, NETosis, and microvascular thrombosis. The resulting clinical injury therefore reflects the combined effects of bubble generation, bubble transport and retention, local hemodynamics and blood rheology, and downstream thromboinflammatory responses.

Experimental models reproduce different portions of this continuum and should be interpreted according to the pathological stage they actually model. Whole-animal decompression models preserve tissue inert-gas kinetics, bubble formation, and systemic disease phenotypes, whereas whole-blood decompression systems provide controlled access to decompression-induced bubble genesis. In contrast, most endothelial-cell, isolated-vessel, and microfluidic platforms use pre-existing or externally generated bubbles that are introduced directly into the experimental system. These models are therefore most informative for studying post-formation bubble behavior and biological responses, including bubble transport, vascular retention or obstruction, endothelial injury, thrombosis, and inflammation. These approaches are complementary rather than interchangeable. Their combined use provides a mechanistic framework for identifying candidate diagnostic biomarkers, evaluating preventive and therapeutic strategies, and defining the biological consequences of intravascular bubbles. Future progress will depend on integrating pressure control, physiologically relevant blood properties, humanized microvasculature, and immune and coagulation components within experimental systems that more closely reproduce the complete DCS sequence. Such integration may ultimately improve the translation of bubble biology into more reliable prevention, diagnosis, and treatment of DCS.

## Figures and Tables

**Table 2:** Comparative features of in vitro and ex vivo platforms using decompression-generated or directly introduced gas bubbles.

Model/Platform	Strengths	Limitations	Controllable Variables	Core Readouts	Primary Applications	Bubble Origin/Processes Modeled	Refs.
Static endothelial-cell bubble exposure	Simple and scalable; compatible with primary or human endothelial cells; strong mechanistic resolution; amenable to inhibitors, siRNA, and drug screening	No physiological shear; limited blood–cell and immune interactions; often uses large or prolonged bubble exposure; single-vessel-bed bias	Cell type; bubble size or gas volume; exposure duration; recovery time; pharmacological or genetic perturbation	Cell viability and apoptosis; EMP release; ROS/NO balance; TEER/ECIS; ICAM-1, VCAM-1, ET-1, IL-1β, TNF-α; autophagy and HSF-1/HSP signaling	Endothelial injury mechanisms; EMP biogenesis; oxidative stress; autophagy; mechanistic qualification of candidate endothelial biomarkers; evaluation of HBO pretreatment/preconditioning and other endothelial-protective interventions	Externally generated bubbles; post-formation endothelial injury	[7,64,68,72,87,88]
Single-microbubble cell-contact systems	Single-cell precision; physiologically relevant microbubble size; defined contact force, duration, and frequency; real-time imaging of target and neighboring cells	Low throughput; technically demanding; lacks flow, blood cells, and systemic inflammatory inputs	Bubble diameter; contact force; contact time; contact number; target-cell type; channel or probe geometry	Intracellular Ca^2+^ dynamics; cell deformation; Ca^2+^ wave propagation; ATP- and gap-junction-dependent signaling	Mechanotransduction; Ca^2+^ signaling; cell–cell communication; mechanosensitive-channel screening	Externally generated single bubbles; post-formation bubble–cell interaction	[80,95]
Ex vivo vessel-ring or vessel-segment assays	Preserves intact vascular wall architecture; separates vascular function from systemic confounders; enables pharmacological dissection of endothelial and smooth-muscle pathways	Limited immune and blood-component interactions; simplified pressure/shear environment; usually one vascular bed; direct bubble perfusion often uses buffer rather than blood	Vessel bed; bubble density and duration; intraluminal pressure/flow; vasoactive agonists; channel, ROS, or NO-pathway inhibitors	ACh-induced endothelial relaxation; SNP-induced smooth-muscle relaxation; PE contraction; EC_50_/Emax; NO, PGI_2_, EDHF, TRPV4/IP_3_R/IKCa and ROS pathway contribution	Direct bubble effects on vascular reactivity; endothelial versus smooth-muscle dysfunction; ion-channel and oxidative-stress mechanisms	Externally perfused bubbles; post-formation vascular injury and dysfunction	[92]
Whole-blood decompression models	Retains native blood cells, plasma proteins, lipids, and coagulation components; more closely preserves physiological hematocrit, viscosity, and gas–blood interfacial composition; captures inter-individual variability; visualizes decompression-induced phase transitions	Static and non-endothelialized; no physiological vascular geometry or shear; anticoagulation, temperature, and sample handling may alter rheological and interfacial properties; bubble size often incompletely quantified; mechanisms require in vivo or chip validation	Final pressure/vacuum level; decompression rate; temperature; surface roughness; donor hematocrit; plasma composition; anticoagulant; blood viscosity/rheological properties	Bubble nucleation threshold; bubble density and morphology; plasma phase transition; evaporation rate; inferred acoustic softening; Sanal-flow choking metrics	Physical basis of bubble nucleation and growth; blood-phase transitions; decompression susceptibility; candidate blood-biomarker discovery under controlled decompression; material-surface effects on nucleation	Decompression-induced in situ bubble formation; bubble nucleation and growth	[63]
Endothelialized microfluidic and vascular-chip models	Reconstitutes flow and shear; enables real-time visualization; supports human endothelial cells, blood perfusion, TEER, multicellular co-culture, and 3D ECM integration; compatible with controlled bubble generation	Incomplete immune and neurohumoral complexity; many channels remain rectangular; PDMS drug absorption; limited standardization and throughput; capillary-scale networks remain challenging; physiological blood rheology and gas–blood interfacial chemistry are often incompletely reproduced or reported.	Channel geometry; shear stress and flow rate; bubble size/frequency; hematocrit; apparent viscosity/rheological model; plasma-protein or surfactant composition; interfacial tension; blood or media composition; endothelial and stromal cell types; ECM stiffness; pharmacological/genetic perturbation.	Bubble motion, deformation, splitting, arrest, and clearance; platelet/fibrin deposition; neutrophil adhesion, transmigration, and NETosis; TEER/permeability; Ca^2+^ signaling; μPIV flow fields; film thickness and bubble residence time; Reynolds and capillary numbers.	Bubble transport and embolization; bubble-induced thrombosis; endothelial barrier failure; mechanistic evaluation of candidate vascular biomarkers under defined bubble and flow conditions; P-selectin/PSGL-1/NOX2/PAD4 signaling; drug screening; humanized and patient-specific modeling	Predominantly externally/mechanically generated bubbles; post-formation transport, embolization, thrombosis, and endothelial injury	[7,9,80,93,94,96,99,100,101]

Table note: Abbreviations: ACh, acetylcholine; DCS, decompression sickness; ECIS, electric cell–substrate impedance sensing; ECM, extracellular matrix; EDHF, endothelium-derived hyperpolarizing factor; EMP, endothelial microparticle; ET-1, endothelin-1; HBO, hyperbaric oxygen; HSP, heat-shock protein; HSF-1, heat-shock factor 1; ICAM-1, intercellular adhesion molecule-1; IKCa, intermediate-conductance Ca^2+^-activated K^+^ channel; IP_3_R, inositol 1,4,5-trisphosphate receptor; MPS, microphysiological system; NETosis, neutrophil extracellular trap formation; NO, nitric oxide; PGI_2_, prostacyclin; PE, phenylephrine; ROS, reactive oxygen species; SNP, sodium nitroprusside; TEER, trans-endothelial electrical resistance; TRPV4, transient receptor potential vanilloid 4; VCAM-1, vascular cell adhesion molecule-1; μPIV, micro-particle image velocimetry.

## Data Availability

No new data were created or analyzed in this study. Data sharing is not applicable to this article.

## Abbreviations

The following abbreviations are used in this manuscript:

ACh	Acetylcholine
AHSs	Active hydrophobic spots
ATA	Atmospheres absolute
ATP	Adenosine triphosphate
C3a	Complement component 3a
C3b	Complement component 3b
C3bc	Complement component 3b/c fragment
C4a	Complement component 4a
C5a	Complement component 5a
CK-MM	Creatine kinase-MM
Cit-H3	Citrullinated histone H3
DCS	Decompression sickness
DPPC	Dipalmitoylphosphatidylcholine
EB	Eftedal–Brubakk
EC50	Half-maximal effective concentration
ECIS	Electric cell–substrate impedance sensing
ECM	Extracellular matrix
EDHF	Endothelium-derived hyperpolarizing factor
Emax	Maximal effect
EMP	Endothelial microparticle
EMPs	Endothelial microparticles
ET-1	Endothelin-1
FXII	Coagulation factor XII
GSDMD	Gasdermin D
H&E	Hematoxylin and eosin
HBO	Hyperbaric oxygen
HPMECs	Human pulmonary microvascular endothelial cells
HSF-1	Heat shock factor 1
HSP	Heat shock protein
HUVECs	Human umbilical vein endothelial cells
ICAM-1	Intercellular adhesion molecule-1
IKCa	Intermediate-conductance calcium-activated potassium channel
IL-1β	Interleukin-1 beta
IL-6	Interleukin-6
IL-18	Interleukin-18
iNOS	Inducible nitric oxide synthase
IP3R	Inositol 1,4,5-trisphosphate receptor
IPAVA	Intrapulmonary arteriovenous anastomoses
iPSCs	Induced pluripotent stem cells
MDA	Malondialdehyde
MPO	Myeloperoxidase
MyD88	Myeloid differentiation primary response 88
NETs	Neutrophil extracellular traps
NF-κB	Nuclear factor kappa B
NLRP3	NLR family pyrin domain containing 3
NO	Nitric oxide
NOX2	NADPH oxidase 2
PAD4	Peptidylarginine deiminase 4
PAVF	Pulmonary arteriovenous fistulas
PDMS	Polydimethylsiloxane
PE	Phenylephrine
PFO	Patent foramen ovale
PGI2	Prostacyclin
PMVECs	Rat pulmonary microvascular endothelial cells
PKCα	Protein kinase C alpha
PSGL-1	P-selectin glycoprotein ligand-1
ROCK	Rho-associated kinase
ROS	Reactive oxygen species
RV	Right ventricle
sGPV	Soluble glycoprotein V
siRNA	Small interfering RNA
SNP	Sodium nitroprusside
TEER	Transendothelial electrical resistance
TLR9	Toll-like receptor 9
TNF-α	Tumor necrosis factor alpha
TRPV	Transient receptor potential vanilloid channel
TRPV4	Transient receptor potential vanilloid 4
TXNIP	Thioredoxin-interacting protein
VCAM-1	Vascular cell adhesion molecule-1
WSS	Wall shear stress
